# Circular RNA circ_0002137 regulated the progression of osteosarcoma through regulating miR‐433‐3p/ IGF1R axis

**DOI:** 10.1111/jcmm.16166

**Published:** 2021-02-23

**Authors:** Meng Zhang, Guang‐Yang Yu, Gang Liu, Wei‐Dong Liu

**Affiliations:** ^1^ Department of Orthopedic The Affiliated Huaian NO. 1 people's Hospital of Nanjing Medical University Huaian China

**Keywords:** circRNA, hsa_circ_0002137, IGF1R, miR‐433‐3p, osteosarcoma

## Abstract

Current clinical treatment targeting osteosarcoma (OS) are limited for OS patients with pulmonary metastasis or relapse, which led to high mortality (70%‐85%) for advanced osteosarcoma patients. Although ongoing efforts have been made to illustrate the mechanisms of tumorigenesis and progression in OS; however, it was far for us to learn a comprehensive molecular mechanism implies in OS development. In our study, we implicated a circRNA hsa_circ_0002137, which was higher expressed in osteosarcoma tumours compared with paracancerous tissue. The dysregulated expression pattern was also found in osteosarcoma cell lines. The role of circ_0002137 was explored via down‐ or up‐regulated experiments. It was proved that down‐regulation of circ_0002137 suppressed the progress of OS, including cell invasion, cell cycle and cell apoptosis. Furthermore, the correlation between circ_0002137 and miR‐433‐3p was predicted using bioinformatic tools and verified utilizing RNA pull‐down assay and luciferase reporter assay. Interestingly, we found that the inhibitory effect of circ_0002137 on OS was dependent of insulin‐like growth factor‐1 receptor (IGF1R). In conclusion, it was demonstrated that circ_0002137 could restrain the progression of OS through regulating miR‐433‐3p/IGF1R axis, providing a comprehensive landscape of circ_0002137 in the generation and development of OS.

## 1 INTRODUCTION

Osteosarcoma, one of the most frequent primary malignant tumours, is regarded as a life‐threatening solid malignancy of bone for children and adolescents predominantly. Osteosarcomas originates from primitive mesenchymal bone cells and usually occurs in long bones instead of soft tissue.^1^, ^2^ The morbidity of osteosarcomas is almost 11 million in paediatric population. The combination of surgery, radiotherapy and chemotherapy improves the prognosis of 60%‐75% patients with OS.^3^, ^4^ However, a large portion of OS patients with pulmonary metastasis or relapse have dismal prognosis on account of untimely diagnosis and drug resistance.^5^ Therefore, it is in urgent need to explore the molecular mechanism of tumour genesis, metastasis and drug resistance, which might provide novel strategy for clinical treatment and improve the outcome of advanced OS patients.

Circular RNAs (circRNAs), one kind of endogenous non‐coding RNA, are characterized by closed continuous loop in shape, and without 5′‐3′ polarity and polyA tail, which give them strong resistance to degradation from enzyme.^6^, ^7^ Though the biological function of circRNAs is ignored in the past, emerging evidences have indicated that circRNAs play an important role in the various processes including physiological development and disease occurrence.^8^, ^9^ The function of circRNAs in the tumorigenesis are multitudinous. Most studied circRNA ciRS‐7 was reported to exhibit high expression profile in different human cancers and could regulate the tumorigenesis by sponging for miR‐7.^10^, ^11^, ^12^ CircFoxo3 has been demonstrated to function as proteins scaffolds through binding to ubiquitin enzyme MDM2 and its substrates p53 to inhibit angiogenesis and cell cycle progression.^13^, ^14^ As to osteosarcoma, a series of differentially expressed circRNAs have been found by microassay expression analysis of circRNAs in OS patients.^15^, ^16^ Among them, circ 2137 was mostly up‐regulated in osteosarcoma tissues, which attracted our attention.^17^ However, the molecular mechanism of circRNA in OS has not been uncovered.

In our study, we implicated a novel circRNA hsa_circ_0002137, whose expression was abnormally up‐regulated in osteosarcoma tumours compared with normal bone tissue. Furthermore, the role of circ_0002137 was investigated via down‐ or up‐regulated experiments and found that circ_0002137 inhibition suppressed cell invasion and cell proliferation of OS cell lines, meanwhile we found knockdown of circ_0002137 could induce more apoptotic OS cells. Furthermore, the correlation between circ_0002137 and miR‐433‐3p was predicted and confirmed through RNA pull‐down assay and dual‐luciferase reporter assay. Interestingly, we found that the inhibitory effect of circ_0002137 on OS was dependent of IGF1R. In conclusion, it was demonstrated that circ_0002137 could restrain the progression of OS through regulating miR‐433‐3p/IGF1R axis, providing a comprehensive landscape of circ_0002137 in the tumorigenesis and development of OS.

## 2 MATERIALS AND METHODS

### 2.1 Clinical samples collection

All clinical samples were collected from 15 untreated OS patients in the Affiliated Huaian NO. 1 people's Hospital of Nanjing Medical University after surgical resection. All patients presented at the hospital. They all were provided written consent authorizing the use of clinical samples for research. Fresh samples were stored in liquid nitrogen. All experiments in this study were approved by ethic committee of the Affiliated Huaian NO. 1 people's Hospital of Nanjing Medical University.

### 2.2 Antibodies

All antibodies used in this study were as following: anti‐IGF1R antibody (Ab39675, Abcam), anti‐GAPDH antibody (MA1‐16757, Thermofisher), horseradish peroxidase‐conjugated secondary antibodies goat anti‐rabbit IgG (H + L) (G‐21234, Thermofisher), goat anti‐mouse IgG (G‐21040, Thermofisher).

### 2.3 Cell culture and transfection

All OS cell lines including MG‐63, U2OS, 143B and G292 and normal bone cells line hFOB 1.19 were purchased from Type Culture Collection of the Chinese Academy of Sciences. Cells were cultured at 37 or 34°C with 5% CO2. Cell medium was Dulbecco's modified Eagle's medium (DMEM, Hyclone) or F12 medium (Hyclone) supplemented with foetal bovine serum (FBS; Gibco) and 10% penicillin and streptomycin (100 U/mL P/S; Gibco). Cell transfection was performed following users guide of Lipofectamine 2000 reagent (Invitrogen).

### 2.4 Reverse transcription‑quantitative polymerase chain reaction (RT‐qPCR) assay

Trizol reagent (Thermo Fisher, Inc) was applied in total RNA extraction from tissues and cell lines. The quality of RNA was determined by using Nanodrop 2000 (Thermo Scientific). If necessary, RNA was incubated with RNase R (Epicentre Biotechnologies) for 15 minutes at 37°C. 3‐5 µg total RNA was reverse‐transcribed to cDNA using PrimeScript First Strand cDNA Synthesis Kit (Takara). The Primes for cDNA synthesis was the mix of oligodT and Random hexamers. Then qPCR was conducted used TB Green® Premix Ex Taq™ GC (Takara) on ABI 7500 Real‑time PCR detection system (Applied Biosystems). The PCR setup was step 1 (95°C 5 minutes); Step 2 (95°C 10 seconds, 60°C 30‐34 seconds, 40 cycles); Melt curve: 95°C 1 minute, 55°C 1 minute, 55‐98°C (10 s/cycle 0.5°C/cycle). The level of RNA was quantified using 2^−ΔΔ^ *^C^* ^q^ method. The Internal control was GAPDH or U6. The relative expression level of RNA was normalized using U6 and GAPDH. The primes were designed using circinteractome and NCBI.^18^, ^19^ GAPDH forward, 5'‑CCGTCTAGAAAAACCTGC C‑3'; and reverse, 5'‑GCCAAATTC GTTGTCATACC‑3'; U6, forward 5'‑CTCGCTTCGGCAGCACA‑3'; and reverse 5'‑AAC GCTTCACGAATTTGCGT‑3'; F: IGF1R, forward 5’‐GTCGAAGAATCGCATCATCA‐3’; and reverse 5’‐GCATCCTGCCCATCATACTC‐3’; miR‐433‐3p forward 5'‐GGAGAAGTACGGTGAGCCTGT‐3’; and reverse 5’ ‐GAACACCGAGGAGCCCATCAT‐3’. Circ_0002137 primer 1: forward 5'‐ CAGAAGGAGGCCTGGTGTAA‐3’; and reverse 5’ ‐ TGCGCACACAGGAACATAAT‐3’ Circ_0002137 primer 2: forward 5'‐ TACTGTCAGAAGGAGGCCTGG‐3’; and reverse 5’ ‐ GCGCACACAGGAACATAATGC‐3’.

### 2.5 Cell proliferation assay

Cell Counting Kit‐8 (CCK8; Dojindo) assay was performed to detect the growth of OS cells. After transfection for 24 hours, 2000 cells were cultivated into 96‐well plate. 10 µL CCK8 reagent was added into each well at different time point then incubated for 2 hours. OD value was recorded at 450 nM on Tecan Infinite Pro M1000 (Tecan).

### 2.6 Transwell assay

After transfection for 48 hours, cells were starved in serum‐free medium for 24 hours. Then 1E5 cells were resuspended with cell medium containing 10% FBS and added into the transwell chamber coated with diluted Matrigel (Yeason, China). Meanwhile, DMEM cell medium containing 20% FBS was added into basolateral chamber. After cultured for 24 hours, the cells were immobilized using 4% polyformaldehyde then stained with 0.5%‐1% crystal violet solution. The images were captured by microscope (Olympus).

### 2.7 Migration assay

Cell migration was detected using wound‐healing assay. Transfected cells were added into 6‐well plate at the density of 1.5E5 cells per well. After being cultured for 12 hours, a straight scratch was scraped using pipette tips (200 µL) in each well and scratch position was marked using dark line. The images were captured at 24 and 48 hours, respectively. Image J software (NIH) were applied to calculate the ratio of wound‐healing area.

### 2.8 RNA pull‐down assay

The RNA pull‐down assay using biotinylated RNA was conducted as previous study described by wang et al.^20^ 1E7 OS cells were washed with PBS followed with being harvested and lysed in a lysis buffer [(20 mM Tris, pH 7.5, 200 mM NaCl, 2.5 mM MgCl2, 0.05% Igepal, 60 U/mL Superase‐In (Ambion), 1mM DTT, protease inhibitors (Roche)). The cell lysates were centrifuged for reserve. Biotinylated circRNA and control probe was incubated with M‐280 streptavidin magnetic beads (Sigma). Before incubation, the beads were coated with RNase‐free BSA and yeast tRNA (Sigma) to prevent unspecific binding with RNA. Then the biotined‐RNA coated beads were incubated with cell lysate at 4°C for 3 hours on shaking table and washed twice. The RNA complexes bound to the beads were extracted with RNeasy mini Kit (Qiagen), then qRT‐PCR assay was performed to qualify the level of RNA.

### 2.9 Flow cytometry

Transfected cells were collected and washed by PBS. For cell cycle distribution, suspended cells were dealt with 70% ethanol (500 μL) and fixed at 4°C overnight. Then, RNase A (Sigma) was added into cell suspension for 20 minutes. Cell cycle was detected with FACS flow cytometry (BD). For cell apoptosis detection, cell suspension was treated with annexin V and propidium iodide dye (PI) (Sigma), then apoptotic cells were calculated by flow cytometry.

### 2.10 Dual‐luciferase reporter assay

The relationship between circ_0002137 and miR‐433‐3p/IGF1R was determined by dual‐luciferase reporter assay. The luciferase reporter vectors with wildtype or mutant 3’‐UTR of circ_0002137 or IGF1R were constructed. Cells were co‐transfected with miR‐433‐3p mimics or miR‐NC using Lipo3000. After 48 hours, the luciferase activity was determined using a kit called dual‐luciferase reporter assay (Promega).

### 2.11 Animal model

4 weeks‐old nude mice were purchased from Shanghai Model Organisms Center (Shanghai, China). 1E7 stable transfected OS cell suspension MG63 or U2OS were subcutaneously injected into the lower dorsal flank on mice. There were six mice per group. After injection, the tumour volume was measured and calculated each 5 days until 5 weeks. Then the animals were sacrificed and nodules were collected and weighed. All animals’ related experiments were approved by ethical committee in the Affiliated Huaian NO. 1 people's Hospital of Nanjing Medical University.

### 2.12 Statistical analysis

GraphPad Prism 6.0 (GraphPad software) was used to make statistics. For the comparison of two group, *F* test was used to compare variances and Kolmogorov‐Smirnov test was used to test Gaussian distribution. If both of *P* values were more than 0.10, unpaired *T*‐ test was used. If the data didn't meet the condition, non‐parametric was used. When more than two groups are compared, Gaussian distribution was test using Kolmogorov‐Smirnov test, only the *P* value >.10, we thought the data was normal distribution. Meanwhile, the homogeneity of variance was tested using *F* test. One‐way‐ANOVA or non‐parametric test was chosen based the result of Kolmogorov‐Smirnov test and *F* test. If one‐way ANOVA was applied, multiple comparisons were tested using Turkey test. All data were represented as mean ± SD. *P* value was indicated using asterisks. Only *P* ≤ .05 was considered statistically significant.

## 3 RESULTS

### 3.1 Up‐regulated expression pattern of Circ_0002137 is identified in OS patients

**FIGURE 1:**
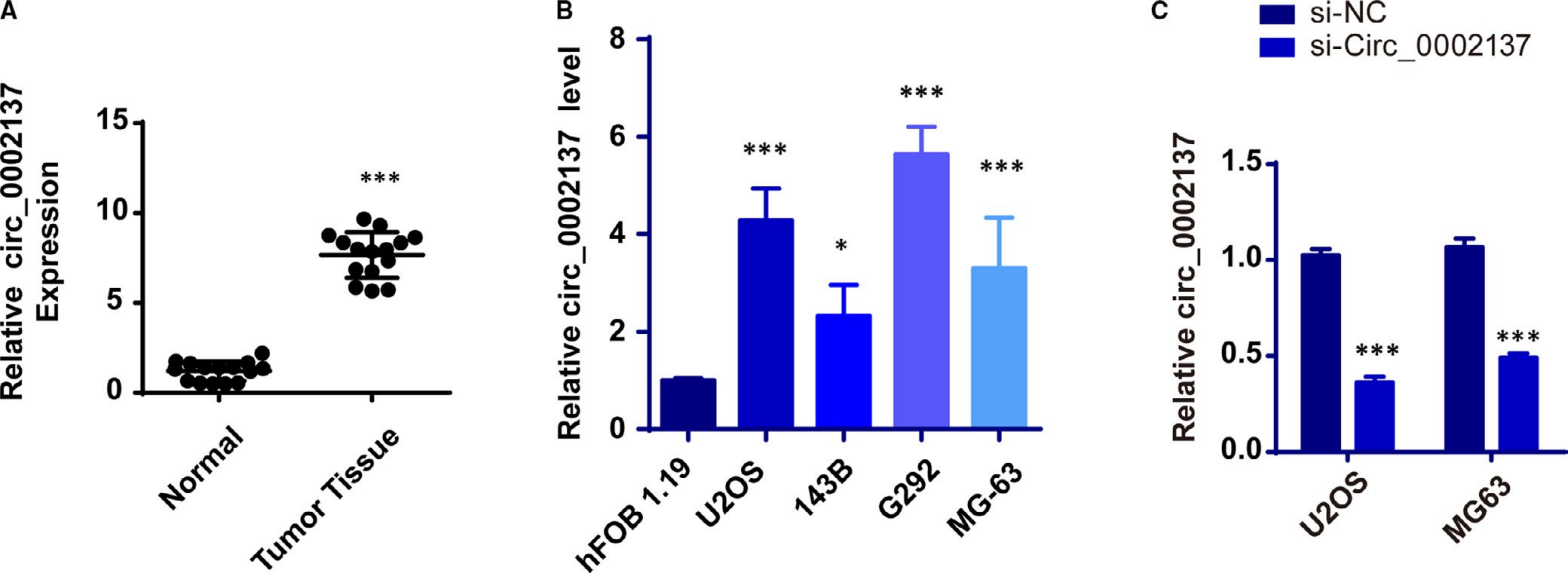
Circ_0002137 is highly expressed in OS patients. A, qRT‐PCR assay determined relative circ_0002137 expression in OS tissues and adjacent non‐tumour tissues, ***P < .001. B, Relative circ_0002137 expression in OS cell lines (U2OS, 143B, G292 and MG‐63) and normal bone cell hFOB 1.19, ***P < .001. C, qRT‐PCR was analysed for circ_0002137 expression in U2OS and MG‐63 cells transfected with si‐circ0002137, ***P < .001

To order to search for potential biomarkers in osteosarcoma, the RNA sequencing was performed by Zhao and their finding indicated circular RNA circ_0002137 was differentially expressed in OS tissue and paired adjacent tissues from OS patients.^17^ Subsequently, our study demonstrated that circ_0002137 was remarkedly up‐regulated in OS patients compared with surrounding non‐tumour tissue using real‐time RT‐PCR (Figure 1A). Meanwhile, abnormal expression pattern of circ_0002137 was also identified in the transcriptional profiling of OS cell lines (U2OS, 143B, SJSA1 and MG‐63) and normal bone cell line (hFOB 1.19) (Figure 1B). To explore the underlying role of circ_0002137 in OS, we constructed the plasmids to interfere the expression of circ_0002137 in OS cell lines and rechecked the efficiency of RNA interference. The result showed that si‐ circ_0002137 reduced the level of circ_0002137 significantly compared with si‐NC group (Figure 1C).

### 3.2 Down‐regulation of Circ_0002137 retrained the proliferation of OS cell lines

**FIGURE 2:**
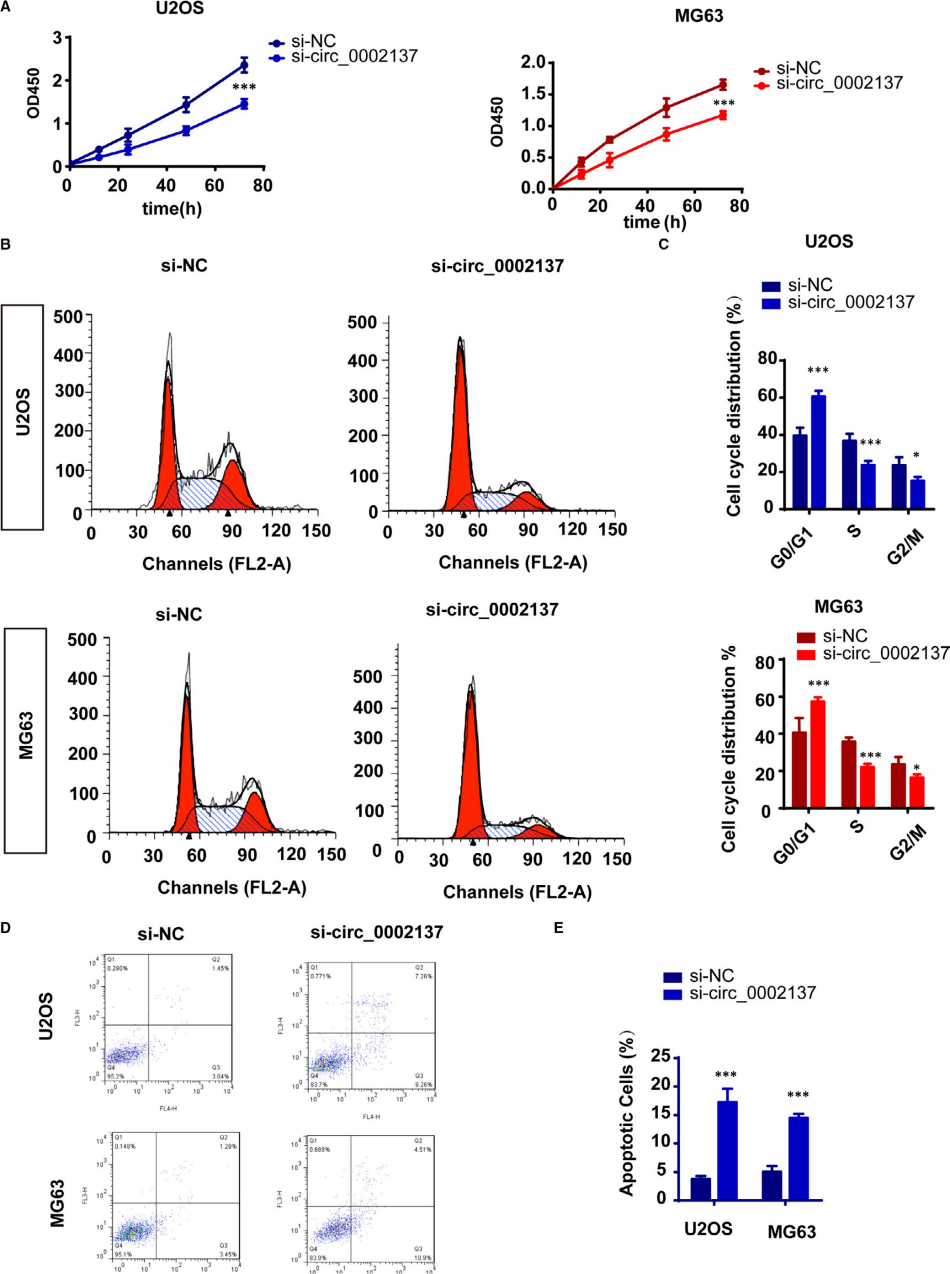
Circ_0002137 inhibition retrained the proliferation of OS cell lines. A, Cell proliferation was detected using CCK8 assay on OS cell lines U2OS and MG‐63 after transfected with si‐circ0002137, ***P < .001. B‐C, Cell cycle arrested was observed using flow cytometry. Si‐circ0002137 group exhibited G0/G1 arrested compared with si‐NC group. ***P < .001, *P < .05. D, Cell apoptosis were determined through staining apoptotic cells with Annexin V‐FITC and PI, ***P < .001

With the purpose of demonstrating whether circ_0002137 could play a role in the tumorigenesis of OS, we conducted the CCK8 assay and found that knockdown of circ_0002137 decelerated the growth of OS cell lines (Figure 2A). To further investigate the negative effect on proliferation caused by circ_0002137, we performed the flow cytometry analysis. The result showed that circ_0002137 inhibition arrested OS cell line in G0/G1 phase (Figure 2B,C). Meanwhile, apoptotic cells were also detected through staining annexin V and PI. The data indicated that knockdown of circ_0002137 promoted the cell apoptosis of OS cell lines (Figure 2D,E). In conclusion, those findings demonstrated that circ_0002137 inhibition suppressed cell proliferation and enhanced cell apoptosis of OS cell lines.

### 3.3 Circ_0002137 inhibition suppressed the metastasis and invasion of OS line

**FIGURE 3:**
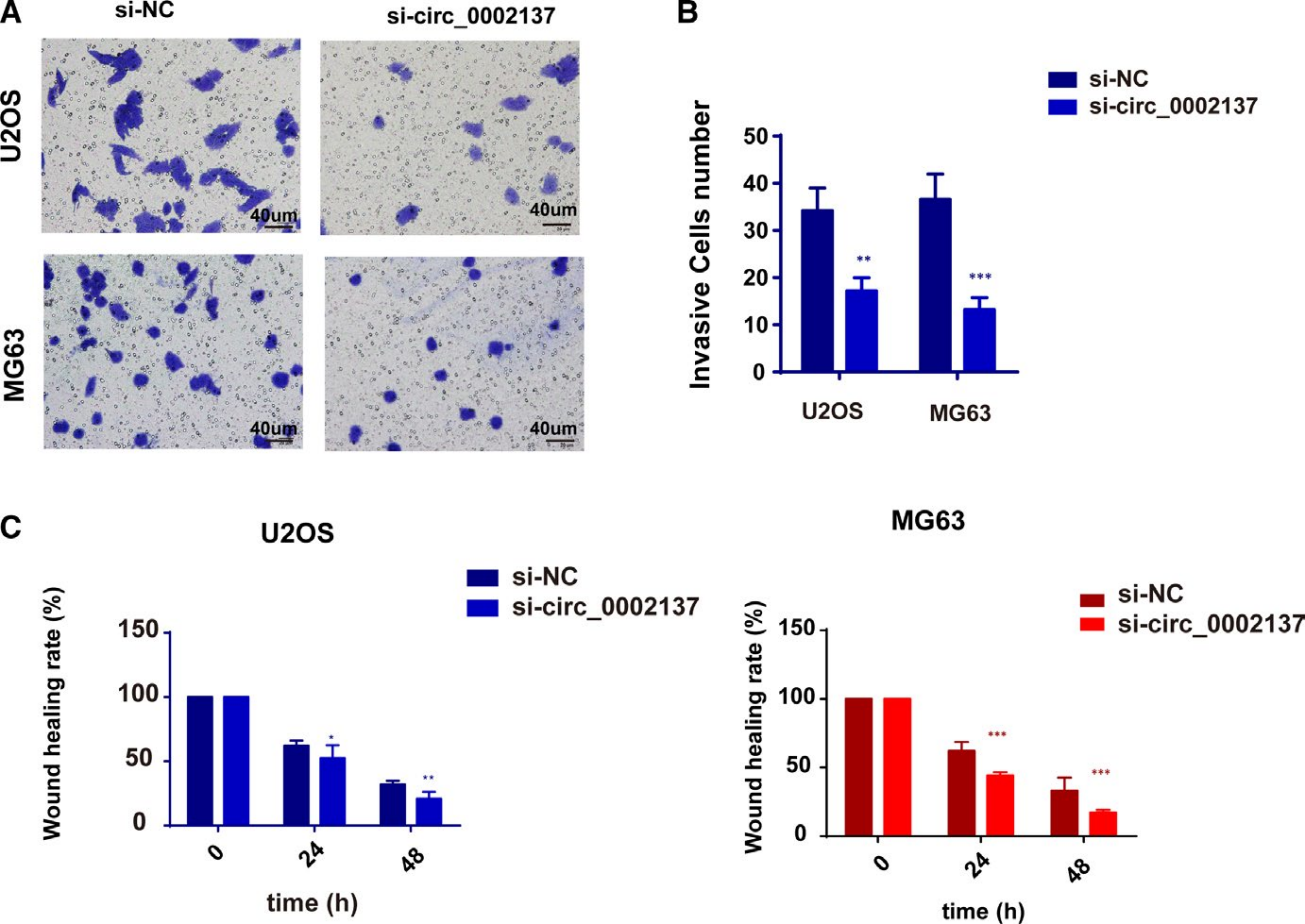
Circ_0002137 inhibition suppressed the metastasis and invasion of OS line. A‐B, Circ_0002137 inhibition reduced the invasion ability of OS cells through transwell assay. ***P < .001; **P < .01. C, Down‐regulation of circ_0002137 decelerated the speed of wound healing in OS cells. ***P < .001; **P < .01; *P < .05

It was known that pulmonary metastasis or relapse usually took place on advanced OS patients, which led to unfavourable prognosis and high mortality. Therefore, it was important to detect whether circ_0002137 had therapeutic potential on inhibiting the metastasis of OS cell line. For this purpose, transwell assay showed that down‐regulation of circ_0002137 significantly decreased the invasion ability of OS cell lines (Figure 3A,B). Moreover, we conducted wound‐healing assay to determine cell migration of OS cells in si‐circ_0002137 group, result showed the migration ability of cells was reduced in si‐circ_0002137compared with si‐NC group (Figure 3C). To sum up, these results revealed circ_0002137 might be the inhibitor of the migration of OS tumour cell lines.

### 3.4 Knockdown Circ_0002137 Suppresses Tumorigenesis of OS in Xenograft Model

**FIGURE 4:**
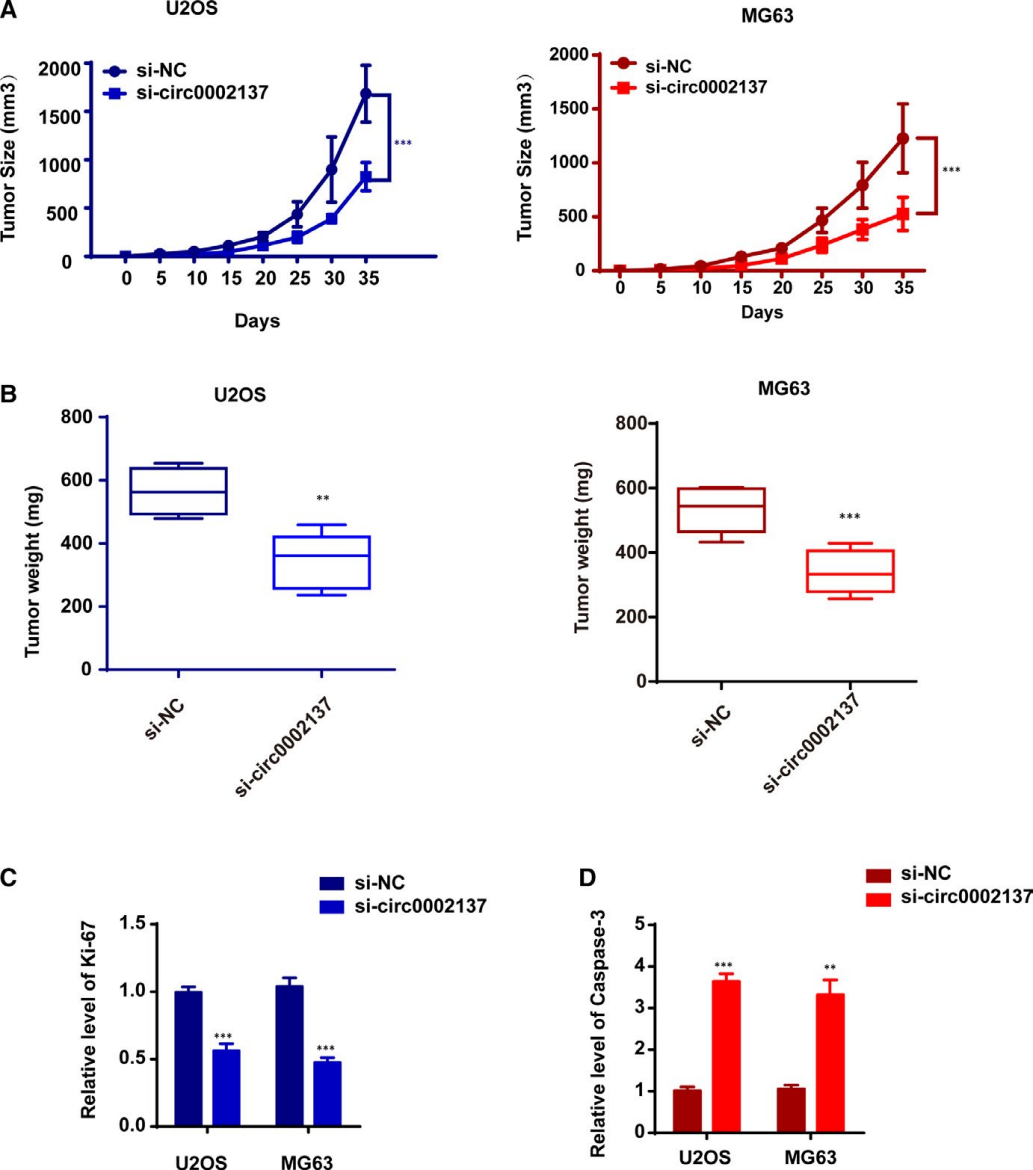
Circ_0002137 Knockdown Suppresses Tumorigenesis of OS in Xenograft Model. A, Tumour growth curves of circ_0002137 inhibitor and si‐NC transfected U2OS cells and MG‐63 in nude mice. ***P < .001. B, The graph of tumour weight indicated circ_0002137 could suppressed the tumour growth in OS model. ***P < .001; **P < .01. C, D, qRT‐PCR was used to evaluate the mRNA level of Ki‐67 and caspase 3 in nodules. ***P < .001; **P < .01

Based on those data in vitro, we postulated that circ_0002137 might have influence on the tumour activity in vivo. Thus, OS cell lines were injected subcutaneously into nude mice after being transfected with si‐circ_0002137 and si‐NC to establish Xenograft model. Results revealed that circ_0002137 suppression significantly retarded tumour growth in vivo in accordance with tumour growth curve and tumour weight (Figure 4A,B). Additionally, knockdown of circ_0002137 inhibited the level of Ki67 and promoted the expression of caspase 3 in si‐circ_0002137 expressing nodules (Figure 4C,D). Therefore, it was demonstrated that circ_0002137 inhibition could restrain the OS’s development in vivo.

### 3.5 Circ_0002137 bond to miR‐433‐3p in OS cell line

**FIGURE 5:**
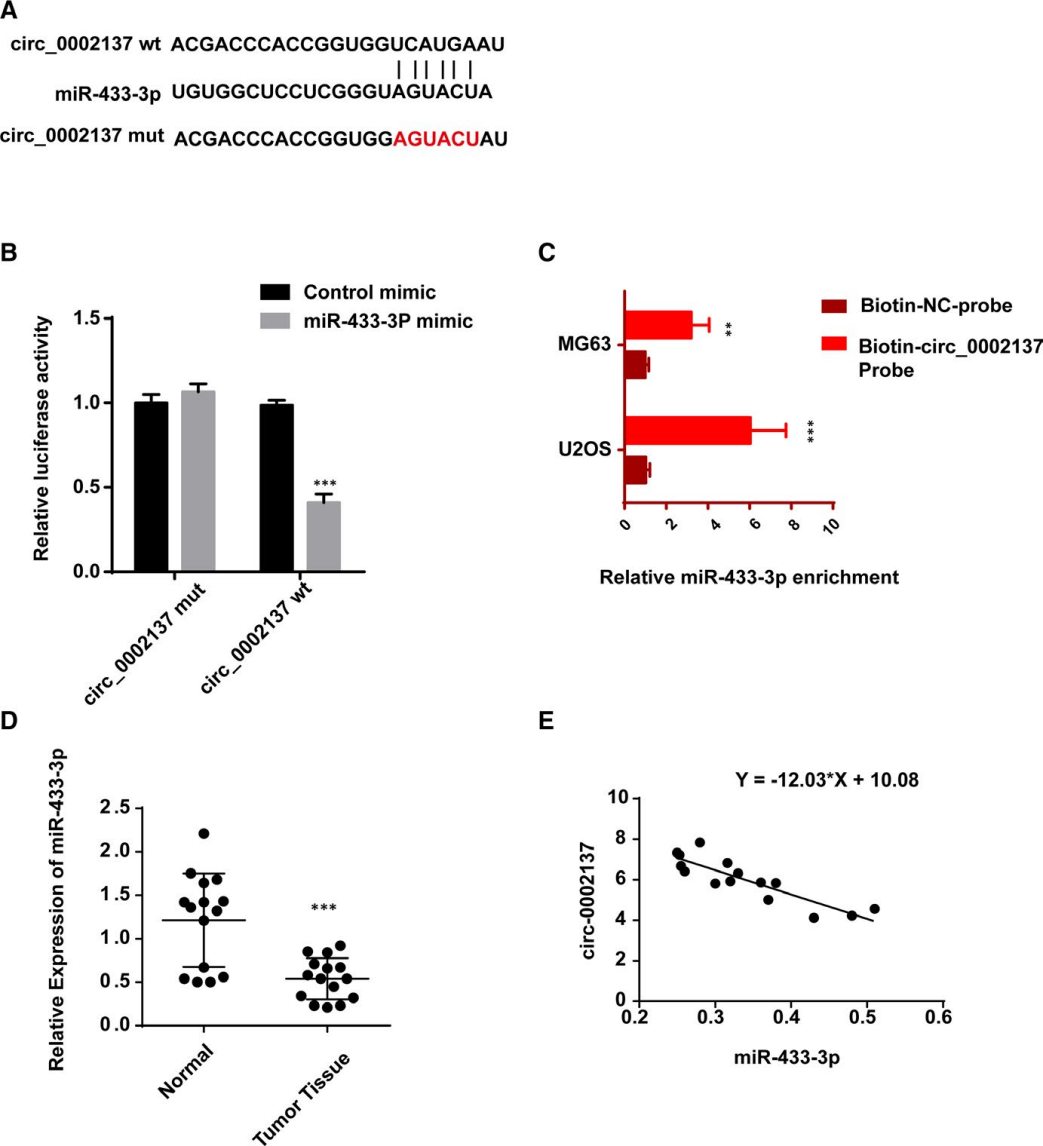
Circ_0002137 bond to miR‐433‐3p in OS cell line. A, Predicted binding sites between circ_0002137 and miR‐433‐3p using circInteractome (NIH, USA). B, Dual‐luciferase reporter assay was conducted to detect the interaction between circ_0002137 and miR‐433‐3p. C, RNA pull‐down assay was performed in order to verify the direct binding of circ_0002137 and miR‐433‐3p. D, The result of qRT‐PCR demonstrated higher level of miR‐433‐3p in OS tissue compared with normal tissue. E, The level of miR‐433‐3p was negatively related with circ_0002137

Emerging evidences have proved that circular RNAs might function as miRNAs’ sponger to regulate the transcription of linear RNAs and the expression of related protein. To further study the molecular mechanism of regulatory effect triggered by circ_0002137, we predicted the downstream molecule of circ_0002137 using circinteractome and circBase and found the potential target might be miR‐433‐3p. Putative‐binding sites between circ_0002137 and miR‐433‐3p were shown in Figure 5A. To evaluate the correlation between circ_0002137 and miR‐433‐3p, dual‐luciferase reporter assay was conducted and proved that overexpression of miR‐433‐3p mimics significantly inhibit the activity of circ_0002137 wt‐luc rather than circ_0002137 mut‐luc (Figure 5B). Furthermore, we confirmed the direct interaction between circ_0002137 and miR‐433‐3p through RNA pull‐down experiment (Figure 5C). Subsequently, we detected the mRNA level of miR‐433‐3p in OS tissue. RT‐QPCR data indicated that miR‐433‐3p had lower level than normal tissue rounding tumour (Figure 5D). We also observed circ_0002137 exhibited negative relationship with miR‐433‐3p in OS cell lines (Figure 5E). We detected the level of miR‐433‐3p when up‐regulating circ_0002137 in OS cell lines. Lower miR‐433‐3p was observed in OS cells transfected with si‐circ_0002137, suggesting the negative correlation between circ_0002137 and miR‐433‐3p. Those findings demonstrated that circ_0002137 could bind to miR‐433‐3p directly and regulate the level of miR‐433‐3p in OS cell lines.

### 3.6 MiR‐433‐3p reversed the inhibitory influence caused by circ_0002137 in OS cell lines

**FIGURE 6:**
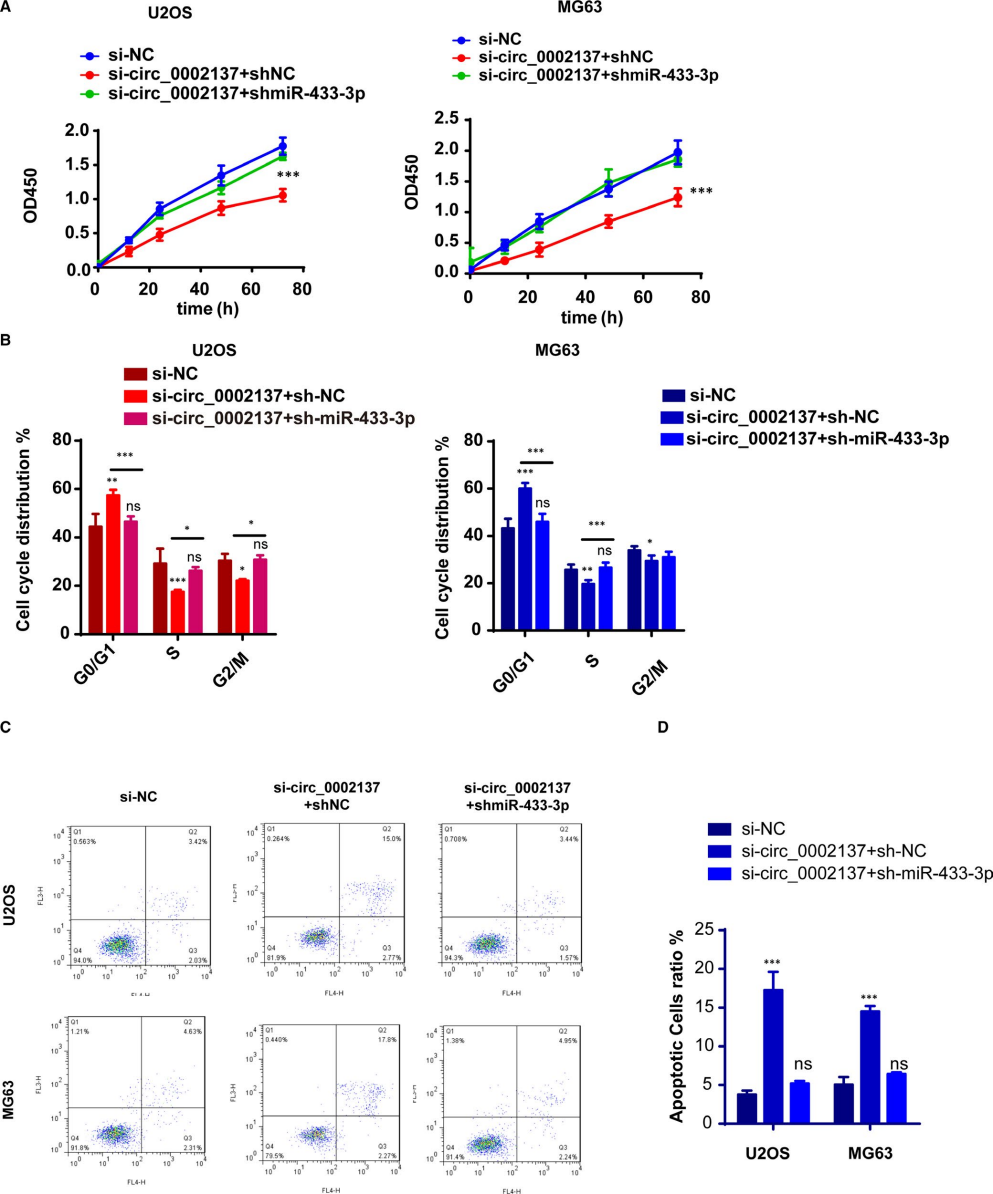
MiR‐433‐3p reversed the inhibitory influence caused by circ_0002137 in OS cell lines. A, Cell proliferation using CCK‐8 indicated that miR‐433‐3p inhibition could restrain the inhibitory effect caused by circ_0002137. B, Cell cycle distribution determined by flow cytometry showed that G0/G1 phase arrest could be rescued by miR‐433‐3p inhibition. C‐D, Cell apoptosis ratio demonstrated that higher ratio of apoptotic cells was reduced in si‐circ_0002137+sh‐miR‐433‐3p group

Next, we explored whether circ_0002137 took part in the development of OS through controlling miR‐433‐3p. To verify the status of cell proliferation, CCK8 assay and cell cycle assay were performed. The final results proved that knockdown of miR‐433‐3p could alleviate the effect caused by circ_0002137 inhibition (Figure 6A,B). Meanwhile, the result of cell apoptosis showed that co‐transfection of si‐circ_0002137 and si‐miR‐433‐3p in OS cells induced less apoptotic cells than OS cells transfected with si‐circ_0002137 alone (Figure 6C,D). In conclusion, we made the conclusion that circ_0002137 modulated the occurrence and progression of OS cell through regulating miR‐433‐3p.

### 3.7 IGF1R as the downstream functional target gene of circ_0002137/ miR‐433‐3p axis

**FIGURE 7:**
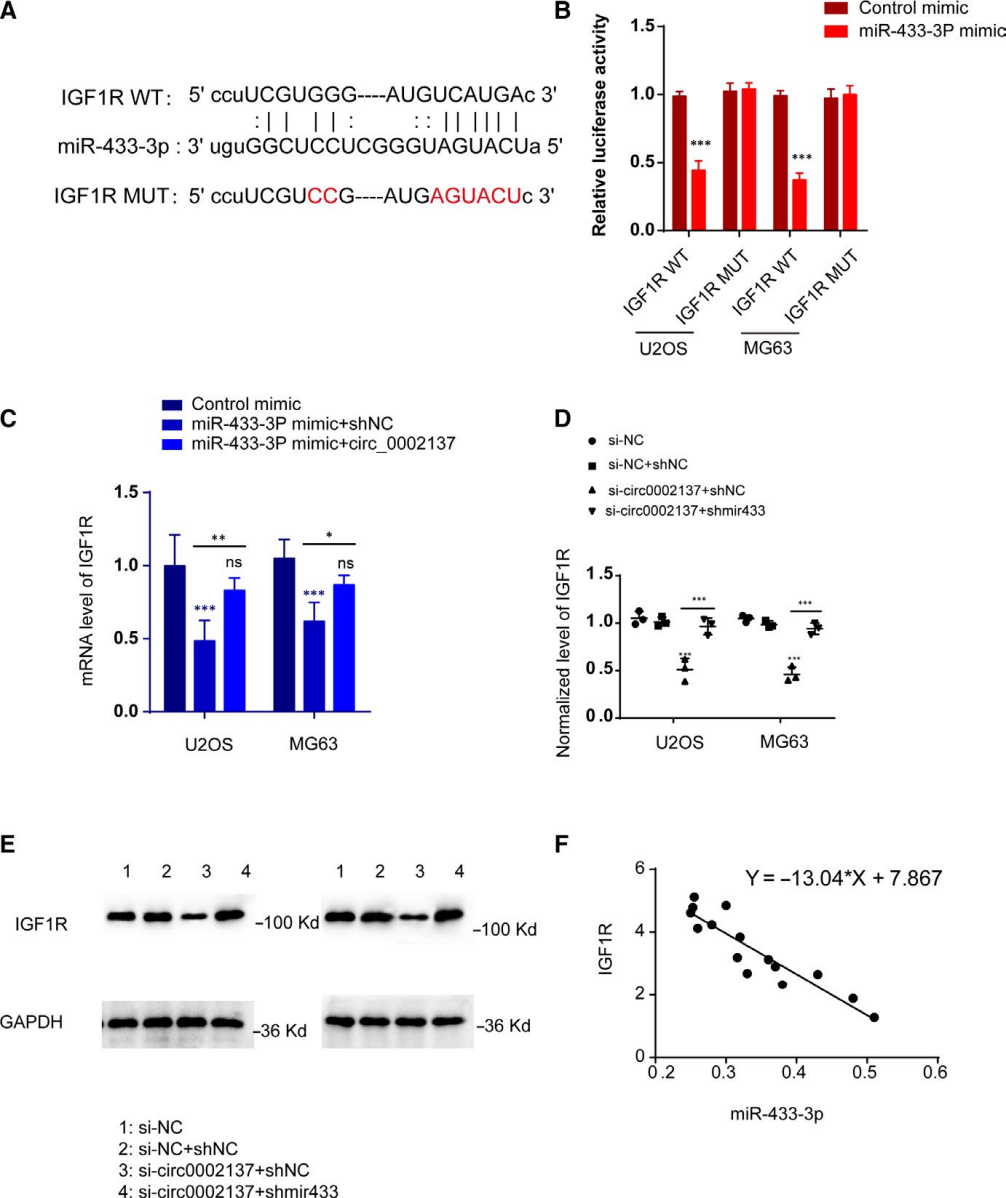
IGF1R as the Downstream Functional Target Gene of circ_0002137/ miR‐433‐3p Axis. A, Predicted binding sites between miR‐433‐3p and IGF1R using Targetscan and Starbase. B, Luciferase reporter assay proved the relationship between miR‐433‐3p and IGF1R. C, QPCR assay indicated mRNA level of IGF1R was reduced by up‐regulation of miR‐433‐3p and the inhibitory effect of up‐regulated miR‐433‐3p could be reversed by co‐expressing circ_0002137. *P < .05; **P < .01; ***P < .001. D‐E, Western blot assay revealed the level of IGF1R was controlled by si‐circ0002137 and shmir433‐3p. F, Negative correlation between IGF1R and miR‐433‐3p was detected by QPCR assay

To explore the underlying mechanism for circ_0002137 in OS, we tried to search for potential partner of circ_0002137/miR‐433‐3p. Firstly, bioinformatic tools (Targetscan and Starbase) were applied to predict IGF1R (insulin‐like growth factor‐1 receptor) might have putative‐binding sites with miR‐433‐3p (Figure 7A). Subsequently, luciferase assay indicated that miR‐433‐3p mimic could affect the expression of IGF1R through regulating the activity of IGF1R promoter (Figure 7B). In addition, mRNA level of IGF1R was reduced with transfecting miR‐433‐3p mimic and the inhibitory effect induced by miR‐433‐3p mimic could be alleviated by co‐expressing circ_0002137 (Figure 7C). As we expected, the protein level of IGF1R was determined using western blot. Results proved that IGF1R was also controlled by circ_0002137/ miR‐433‐3p (Figure 7D,E). Moreover, the relationship between IGF1R and miR‐433‐3p exhibited negatively correlated (Figure 7F). In conclusion, we made the conclusion that circ_0002137 could act as sponger of miR‐433‐3p and modulate the occurrence and development of OS through IGF1R.

## 4 DISCUSSION

IGF1R, one kind of transmembrane tyrosine kinase receptor, has been accepted its role in modulating cell differentiation, proliferation and apoptosis in several cancers.^21^, ^22^, ^23^, ^24^, ^25^ Meanwhile, previous studies have demonstrated insulin‐like growth factors IGF1 and IGF2 were the ligands of IGF1R and their interaction could activate two major oncogenic signalling cascades: PI3K pathway and MAPK pathway.^26^, ^27^ Intriguingly, IGF1R overexpression was observed in multiple kinds of tumour tissue, especially in OS patients. In addition, poor prognosis and survival rate was observed among the OS patients with elevated IGF1R.^28^ Thus, IGF1R was considered as an important target for OS treatment.^29^ In present, several clinical trials targeting IGF1R have been launched. Most of ongoing clinical trials were aimed to blocking IGF1R’s function via taking advantage of inhibitors of IGF1R, such as monoclonal antibody cixutumumab and robatumumab.^30^, ^31^ Unluckily, both of their clinical trials were terminated because of lacking approving efficacy.^30^, ^32^ Similar outcomes occurred in the IGF ligand‐neutralizing antibodies: BI836845 and MEDI‐573.^33^, ^34^ By analysing those clinical data from medicine trials, we found that blocking bioactivity of IGF1R or IGF ligands using antibody was not appropriate because antibodies could not eliminate the overexpression of IGF on the surface of tumour cells. Thereby, other formats of IGF1R inhibitor need to be developed.

In our study, we reported a novel modulator of IGF1R: circ_0002137, which was reported to be differentially expressed in OS patients^17^; however, its molecular mechanism was still unclear. Then we found the direct target of circ_0002137 might be miR‐433‐3p, which was identified to be a tumour suppressor in multiple tumours. For instance, the level of miR‐433 was associated with unfavourable prognosis for the overall survival for patients with gastric carcinoma.^35^, ^36^ In addition, miR‐433 also functioned in liver cancer cell through inhibiting cell migration.^37^ Notably, the role of miR‐433‐3p in osteosarcoma also was reported by Mao et al.^38^ In our study, we found miR‐433‐3p might be a crucial partner of circ_0002137. Considered the relationship between miR‐433‐3p and IGF1R, we proposed the hypothesis that circ_0002137 regulated the progression of osteosarcoma through sponging miR‐433‐3p/IGF1R. Even though both circ_0002137 and miR‐433‐3p have ability to inhibit the overexpression of IGF1R on tumour cells, circ_0002137 was more stabilized than miRNAs because of the special structure of circular RNAs. In short, our study about RNAs targeting IGF1R might provide a promising therapeutic strategy for clinical treatment. Though the finding was promising and interesting, there are several challenges lying in the way of turning the idea into reality. To deliver RNA therapeutic agents into tumour cells accurately and avoid off‐target effects of RNA drugs needed to be solved in the future study.

## CONFLICT OF INTEREST

The authors declare that they have no conflict of interest or personal relationships that could have appeared to influence the work reported in this paper.

## AUTHOR CONTRIBUTION

**Meng Zhang:** Data curation (lead); Formal analysis (equal); Investigation (equal); Methodology (equal); Resources (equal). **Guang‐Yang Yu:** Data curation (supporting); Investigation (equal); Methodology (equal); Validation (equal). **Wei‐Dong Liu:** Project administration (equal); Supervision (equal); Writing‐original draft (equal); Writing‐review & editing (equal). **Gang Liu:** Project administration (equal); Supervision (equal); Writing‐original draft (equal); Writing‐review & editing (equal).

## ETHICAL APPROVAL

The study was approved by local Ethics Committee and all animal experiments followed the institutional guidelines for the care and use of animals. The Approval number is IACUC‐1906034.

## Data Availability

The data used to support the findings of this study are available from the corresponding author upon request.
